# First report of a urinary *Pseudomonas juntendi* carrying *bla*_NDM-1_ and *bla*_IMP-15_ co-integrated into the chromosome via *ICE-IS91* and integron*-Tn402*-like transposition modules

**DOI:** 10.3389/fmicb.2026.1724958

**Published:** 2026-01-29

**Authors:** Ziheng Wang, Jie Li, Yingying Li, Zihao Chen, Enze Ren, Peng Zhang

**Affiliations:** 1 Department of Laboratory Medicine, Yijishan Hospital of Wannan Medical College, Wuhu, Anhui, China; 2 Central Laboratory, The First Affiliated Hospital of Wannan Medical College, Wuhu, China; 3 School of Electronics and Information Engineering, Anhui University, Hefei, Anhui, China

**Keywords:** *bla*_IMP-15_, *bla*_NDM-1_, integrative and conjugative element, metallo-*β*-lactamase, multidrug resistance, *Pseudomonas juntendi*, *Tn3926*

## Abstract

**Background:**

*Pseudomonas juntendi* is an emerging opportunistic pathogen first described in 2019, whose antimicrobial resistance mechanisms and clinical significance remain poorly understood. In this study, we report the first urinary isolate of *P. juntendi* (PJ1) co-harboring *bla*_NDM-1_ and *bla*_IMP-15_, and comprehensively analyzed its phylogeny, resistance architecture, and biological characteristics.

**Methods:**

Species identification and phylogenetic placement were determined using whole-genome sequencing and average nucleotide identity analyses. Genomic annotation was applied to resolve the structure of resistance islands. Biofilm formation, stress tolerance, and virulence were assessed through crystal violet staining, environmental stress assays, and *Galleria mellonella* infection models, respectively.

**Results:**

Phylogenomic analysis revealed that PJ1 clustered with isolates from China and Japan, forming an East Asian lineage suggestive of regional dissemination. Genomic analysis showed that PJ1 carries two metallo-*β*-lactamase modules integrated into the chromosome: *bla*_NDM-1_ embedded within an *ICE–IS91* composite island and *bla*_IMP-15_ located in an integron*-Tn402-*like module, representing a mosaic multidrug resistance island. Phenotypically, PJ1 exhibited robust biofilm formation, tolerance to bile salts and hyperosmotic stress, and high virulence in the *G. mellonella* model.

**Conclusion:**

PJ1 represents the first urinary *P. juntendi* isolate carrying both *bla*_NDM-1_ and *bla*_IMP-15_ on a single chromosome. Its composite resistance island and strong colonization capacity suggest that *P. juntendi* may serve as an emerging reservoir for metallo-*β*-lactamase dissemination, posing potential clinical and epidemiological threats.

## 1 Introduction

The genus *Pseudomonas* comprises non-fermentative Gram-negative rods that frequently cause urinary tract and bloodstream infections, particularly in immunocompromised patients or those with indwelling urinary devices (Reynolds and Kollef, 2021). Previous studies have demonstrated that *Pseudomonas aeruginosa* exhibits remarkable antimicrobial resistance and adaptive capacity in urinary tract infections (UTIs), providing valuable insights into the pathogenic potential of other *Pseudomonas* species (Newman et al., 2022). Moreover, the *Pseudomonas putida* group is considered an important reservoir of resistance genes, capable of disseminating various *β*-lactamase determinants via mobile genetic elements, thereby increasing the risk of multidrug resistance (Brovedan et al., 2021). Among them, a novel species within the *Pseudomonas putida* complex, namely *P. juntendi*, was first formally described in 2019 and isolated from clinical specimens obtained in Japan and Myanmar (Tohya et al., 2019). In recent years, increasing reports of clinical isolates of *P. juntendi* have emerged, suggesting that this species is not merely a rare laboratory variant but rather a potentially clinically relevant pathogen.

Among carbapenemases, metallo-*β*-lactamases (MBLs) represent a major mechanism conferring resistance to broad-spectrum *β*-lactams and carbapenems in the genus *Pseudomonas*. Among them, the NDM and IMP lineages have drawn particular attention, as they confer resistance to nearly all *β*-lactam and *β*-lactamase inhibitor combinations (Wu et al., 2019; Salahuddin et al., 2018). Since its first identification in India in 2008, NDM-1 metallo-*β*-lactamase has disseminated widely among various Gram-negative pathogens (Walsh et al., 2011). In recent years, the emergence and nosocomial transmission of NDM-1-producing *P. aeruginosa* strains have been increasingly reported in Europe and the Mediterranean region, highlighting the global spread of MBLs (Pappa et al., 2024). Among IMP variants, IMP-15 has been repeatedly detected among clinical isolates, and its co-occurrence with NDM-type enzymes underscores the pivotal role of horizontal gene transfer in mediating the dissemination of carbapenem resistance (Tran et al., 2021).

Recent studies from China and other regions have indicated that *P. juntendi* can harbor multiple *β*-lactamase genes and exhibit multidrug-resistant phenotypes. A clinical urinary isolate from China was reported to carry *bla*_IMP-1_ with a novel genetic background (Zheng et al., 2022). In addition, genomic analyses have revealed that certain *P. juntendi* strains may simultaneously possess multiple *β*-lactamase genes such as *bla*_IMP-1-like_, *bla*_OXA-1_, and *bla*_VIM-2_ (Jiang et al., 2024). Furthermore, a fecal isolate of *P. juntendi* was recently found to co-harbor *tmexCD–toprJ*, *bla*_NDM-1_, and *bla*_PME-1_, demonstrating this species’ capacity to integrate diverse resistance determinants within a single genome (Liu et al., 2025). It is noteworthy that clinical isolates of *P. juntendi* were often misidentified as *P. putida* in the early stages, which may compromise epidemiological surveillance and delay the recognition of MBL-dominated resistance mechanisms as well as the implementation of appropriate infection control measures (Tohya et al., 2022).

In this context, we report a urinary isolate of *P. juntendi* co-harboring *bla*_NDM-1_ and *bla*_IMP-15_. Although previous studies have described *P. juntendi* strains carrying either NDM or IMP genes individually, the coexistence of both determinants within the same clinical urinary isolate remains exceedingly rare (Zheng et al., 2022; Liu et al., 2025). Through whole-genome sequencing and resistance mechanism analysis, this study aimed to elucidate the species characteristics, genetic environment of resistance genes, and their potential clinical implications. This finding provides new insights for clinical antimicrobial management and offers valuable evidence for infection control and epidemiological surveillance.

## 2 Materials and methods

### 2.1 Clinical specimen collection and bacterial isolation

Urine samples were obtained from a patient with a urinary tract infection (UTI) admitted to a tertiary hospital in Wuhu, Anhui Province, China, following standard aseptic procedures. The specimens were inoculated within 2 h of collection onto blood agar, MacConkey agar, and cetrimide selective agar plates, and incubated at 37 °C for 24 h. Based on colony morphology, suspected isolates were selected for further identification.

### 2.2 Species identification and molecular confirmation

Species identification was initially performed using the Vitek Mass Spectrometry system (bioMérieux, France). To improve taxonomic accuracy, whole-genome sequencing (WGS) was conducted, and the average nucleotide identity (ANI) was calculated using the online platform JSpeciesWS. ANI comparisons were performed between the assembled genome and reference genomes of *Pseudomonas plecoglossicida*, *P. putida*, *Pseudomonas monteilii*, *Pseudomonas fulva*, and *P. juntendi* (Richter et al., 2016). Core-genome SNPs were extracted using Snippy with *Pseudomonas juntendi* PP_2463 (GCA_021560075.1) as the reference genome and were used for phylogenetic analysis, with the maximum-likelihood tree generated in MEGA X under the HKY model with default parameters and 1,000 bootstrap replicates, and visualized using ChiPlot (Xie et al., 2023).

### 2.3 Antimicrobial susceptibility testing

Antimicrobial susceptibility testing was performed using the Vitek 2 Compact system (bioMérieux, France) for routine antibiotics. The susceptibility of ceftazidime-avibactam and eravacycline was determined using the Kirby-Bauer disk diffusion method. The results were interpreted according to the Clinical and Laboratory Standards Institute (CLSI) guidelines (M100, 34th edition). For agents without species-specific breakpoints for *P. juntendi*, interpretations were based on the CLSI criteria for *P. aeruginosa* or on non-species-related breakpoints, as recommended. *Escherichia coli* ATCC 25922 and *P. aeruginosa* ATCC 27853 were used as quality control strains.

### 2.4 Whole-genome sequencing and genome assembly

Genomic DNA of PJ1 was extracted using the NucleoBond® HMW DNA Kit (Macherey-Nagel, Germany). DNA concentration and purity were determined using a Qubit 4.0 fluorometer (Thermo Fisher Scientific, USA) and a NanoDrop spectrophotometer, while DNA integrity was verified by agarose gel electrophoresis. Sequencing libraries were prepared and subjected to paired-end sequencing (PE150) on the MGI DNBSEQ-T7 platform. Raw reads were quality-filtered using Fastp, and genome assembly was performed with Unicycler, followed by polishing with NextPolish (Wick et al., 2017; Hu et al., 2020). The assembled genome was visualized using Circos software (Krzywinski et al., 2009). Use CGO, GO, and KEGG databases for relevant functional classification annotations (Kanehisa and Goto, 2000; Bumm et al., 2002; Harris et al., 2004).

### 2.5 Detection of antimicrobial resistance genes

After genome sequencing and assembly, antimicrobial resistance genes were identified using the Comprehensive Antibiotic Resistance Database (CARD) and ResFinder databases (Alcock et al., 2023; Florensa et al., 2022). Only high-confidence matches were retained, and the genomic locations of key resistance genes were manually verified in the annotated genome.

### 2.6 Genetic context and analysis of mobile genetic elements

Potential plasmid backbones or mobile genetic element (MGE) contexts were further analyzed using the MOB-suite, PLSDB, and ISfinder databases (Robertson and Nash, 2018; Galata et al., 2019; Siguier et al., 2006). Collinearity analysis of antimicrobial resistance genes and their surrounding regions was visualized using Easyfig (Sullivan et al., 2011).

### 2.7 Determination of biofilm formation ability

Diluted bacterial suspensions of PJ1 at the logarithmic growth phase (OD₆₀₀ = 0.6) were added to 96-well polystyrene plates. After incubation, the culture medium was discarded, and the wells were washed three times with PBS before staining with crystal violet solution. After destaining with distilled water, 95% ethanol was added to dissolve the crystal violet, and the OD₅₇₀ value was measured using a microplate reader. Each group included four technical replicates, and the experiment was performed three times.

The biofilm-forming capacity was classified following the criteria described by Stepanović et al. (2007). The cut-off value (ODc) was defined as the mean OD of the negative control plus three standard deviations, and isolates were categorized as non (OD ≤ ODc), weak (ODc < OD ≤ 2 × ODc), moderate (2 × ODc < OD ≤ 4 × ODc), or strong biofilm producers (OD > 4 × ODc).

### 2.8 Stress tolerance assay

We simulated host-like environmental stresses (bile salts, osmotic pressure, and acidity) to evaluate the survival capacity of the strain. Log-phase bacterial cultures were diluted 1:100 in LB medium containing different concentrations of bile salts, NaCl, or HCl and incubated at 37 °C with shaking at 180 r/min. The optical density at 600 nm was measured hourly using a UV spectrophotometer, and growth curves were plotted with GraphPad Prism 10.

### 2.9 Galleria mellonella infection model

Bacterial cultures in the logarithmic phase were diluted to 1 × 10⁶ CFU/mL and 1 × 10^7^ CFU/mL, respectively. The larvae were divided into six groups, with 10 individuals per group. Each larva was injected with 10 μL of bacterial suspension or PBS into the last left proleg. The hypervirulent *Klebsiella pneumoniae* strain NTUH-K2044 was used as the positive control, and PBS served as the negative control. After infection, the larvae were incubated at 37 °C in the dark, and survival was monitored every 12 h. The survival data were used to generate Kaplan–Meier survival curves (Mai et al., 2023).

## 3 Results

### 3.1 Identification and taxonomic classification of the clinical isolate

The patient was a 71-year-old man with longstanding pneumoconiosis, chronic obstructive pulmonary disease, and recurrent pneumothorax, who was admitted on 5 July 2025 for worsening cough and dyspnea. During the hospitalization, he underwent repeated chest drainage and received several courses of broad-spectrum antimicrobial therapy and continuous intravenous treatments over nearly 3 weeks, creating a setting of sustained selection pressure. Against this clinical background, a urine specimen collected during his hospital stay grew a multidrug-resistant *P. juntendi* strain (PJ1).

**Figure 1:**
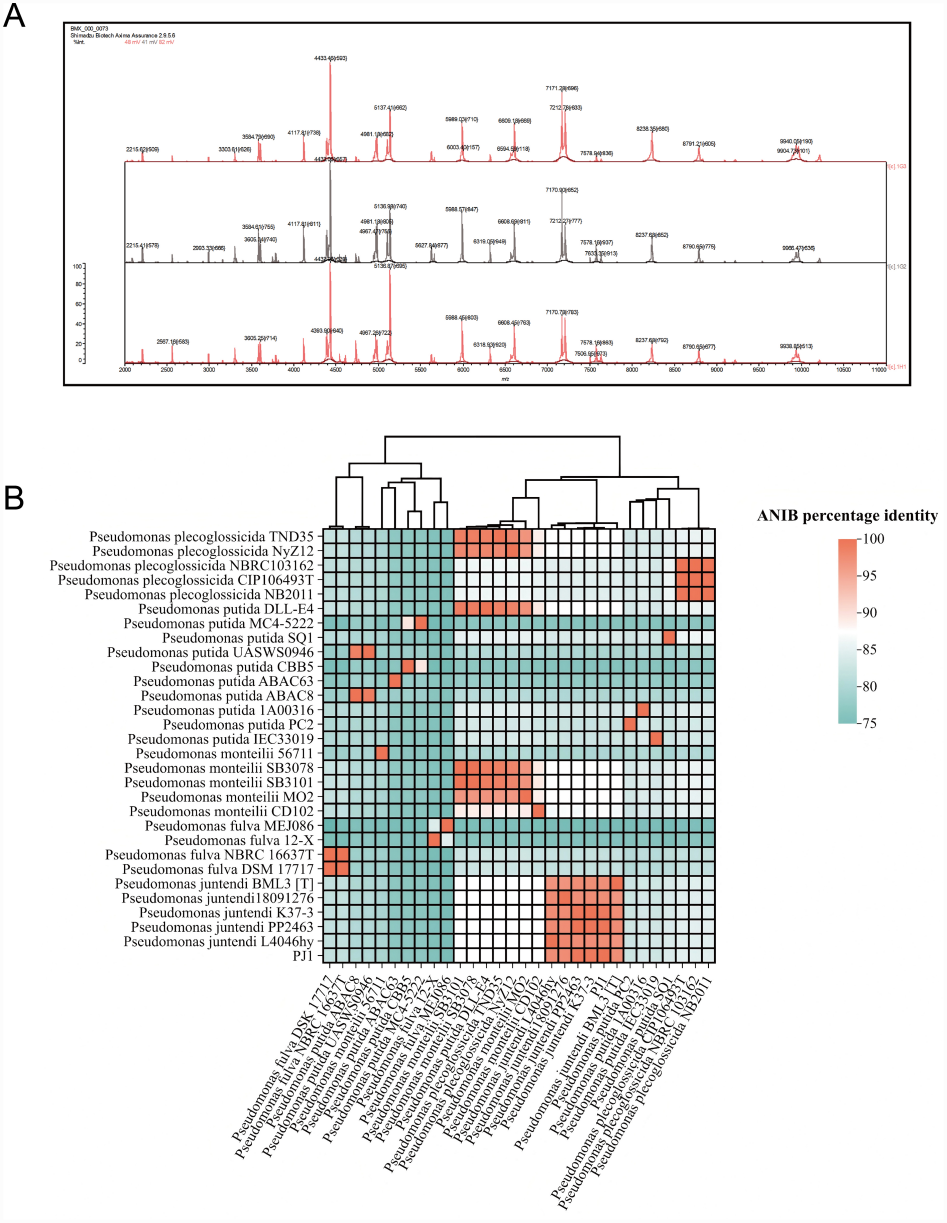
Identification of strain PJ1. **(A)** MALDI-TOF MS analysis of PJ1, performed in triplicate. All three spectra matched the *P. putida* group. **(B)** Heatmap of ANIb scores between PJ1 and 29 reference genomes. Color gradient represents ANIb similarity (from 90% in light yellow to 100% in dark red).

After incubation of the urine specimen on blood agar plates for 18 h, white colonies were observed (Supplementary Figure S1). Species identification using MALDI-TOF MS was performed in triplicate, and all results indicated *P. putida* (Figure 1A). To further clarify the taxonomic assignment, average nucleotide identity (ANIb) analysis based on whole-genome sequencing was conducted. The results showed that the isolate shared 99.63% similarity with *P. juntendi* PP2463 (GCA_021560075.1), and 97.42–99.46% ANIb values with *P. juntendi* reference strains BML3 [T], K37-3, L4046hy, and 18,091,276. As these values exceeded the 95% species delineation threshold, the isolate was designated as *P. juntendi* PJ1. The ANIb values between PJ1 and *P. fulva* (NBRC 16637T) and *P. putida* (DLL-E4) were 81.7 and 87.16%, respectively, while those with *P. monteilii* and *P. plecoglossicida* ranged from 84.5 to 87.0%. Taken together, these results confirmed that PJ1 should be taxonomically classified as *P. juntendi* (Figure 1B).

**Figure 2:**
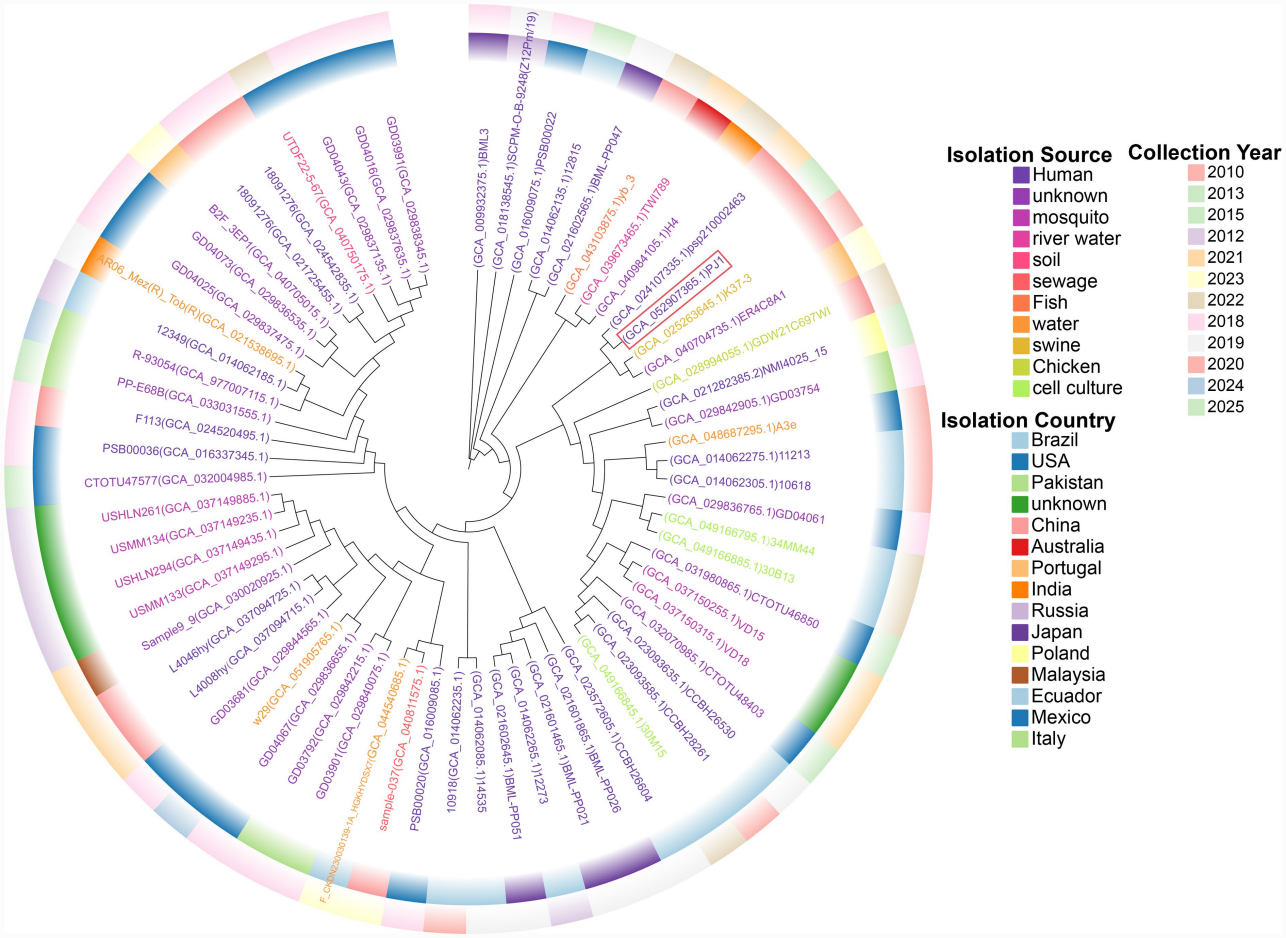
Core-genome SNP-based phylogenetic relationships among all *P. juntendi* isolates deposited in the NCBI database. In the phylogenetic tree, the leaf colors denote different isolation sources, whereas the inner and outer concentric rings indicate the country and year of isolation, respectively.

A core-genome SNP-based phylogenetic tree of all 67 *P. juntendi* isolates currently recorded in the NCBI database was constructed (Figure 2). The results showed that PJ1 clustered within a small clonal group composed of four isolates. Within this cluster, PJ1 showed an almost clonal relationship with the urine-derived isolate PP2463 (GCA_024107335.1) recovered in 2021 from a urinary tract infection patient in Sanmen, China. Core-genome SNP analysis further supported this close relatedness, showing only 4 SNP differences between PJ1 and PP2463. In contrast, the genomic distances to the other two members of the cluster were substantially larger, with 796 SNPs separating PJ1 from the sow vaginal isolate GCA_025263645.1 (Hunan, China, 2020) and 1,154 SNPs from the animal intestinal isolate GCA_040704735.1 (Portugal, 2023). These findings suggest that the lineage represented by PJ1 may have epidemiological relevance in human populations in eastern China and may possess the potential for cross-host transmission involving humans, animals, and environmental reservoirs.

### 3.2 Antimicrobial resistance genes and susceptibility profiles

According to the antimicrobial susceptibility results shown in Table 1, strain PJ1 exhibited a broad-spectrum multidrug-resistant profile. PJ1 showed high-level resistance to *β*-lactam antibiotics, including piperacillin-tazobactam, ceftazidime, cefepime, and cefoperazone-sulbactam, as well as to carbapenems (imipenem, meropenem, and ertapenem). The strain also demonstrated marked resistance to aminoglycosides, fluoroquinolones, trimethoprim-sulfamethoxazole, and tetracyclines. In contrast, PJ1 remained susceptible to tigecycline, eravacycline, and polymyxin B.

**Table 1:** Minimum inhibitory concentrations (MICs) of antimicrobials against *Pseudomonas juntendi* PJ1 and the corresponding resistance genes.

Antibiotic class	Antimicrobial resistance gene(s)	Antimicrobial agent	MIC^a^(μg/mL)
β-Lactams	*bla*_NDM-1_ *bla*_IMP-15_ *bla*_PME-1_ *bla*_CARB-2_	Ticarcillin-Clavulanate	**>128(R)**
		Piperacillin-Tazobactam	**>128(R)**
		Cefoperazone-Sulbactam	**>64(R)**
		^b^Ceftazidime-Avibactam	**R**
		Ticarcillin	**>128(R)**
		Piperacillin	**>128(R)**
		Aztreonam	**>64(R)**
		Ceftazidime	**>64(R)**
		Cefotaxime	**>64(R)**
		Cefpodoxime	**>64(R)**
		Cefepime	**>32(R)**
Carbapenems	*bla*_NDM-1_ *bla*_IMP-15_ *bla*_PME-1_	Imipenem	**>16(R)**
		Meropenem	**>16(R)**
Aminoglycosides	*aadA11* *aph(3′)-Ib* *aph(3′)-VI* *rmtB*	Amikacin	**>64(R)**
		Tobramycin	**>16(R)**
Quinolones	*qnrVC1*	Levofloxacin	**>8(R)**
		Ciprofloxacin	**>4(R)**
		Norfloxacin	**>16(R)**
Tetracyclines	*tet(G)*	Tetracycline	**>16(R)**
		Doxycycline	**>16(R)**
		Minocycline	1(S)
Glycylcyclines		Tigecycline	2(S)
		^b^Eravacycline	S
trimethoprim/sulfonamides	*sul1* *sul2* *dfrA27*	Trimethoprim-Sulfamethoxazole	**>320(R)**
Polymyxins		^b^Polymyxin B	S

a MIC values above the CLSI clinical breakpoints are shown in bold.

b Ceftazidime-Avibactam, Eravacycline and Polymyxin B were tested by the Kirby-Bauer disk diffusion method.

**Figure 3:**
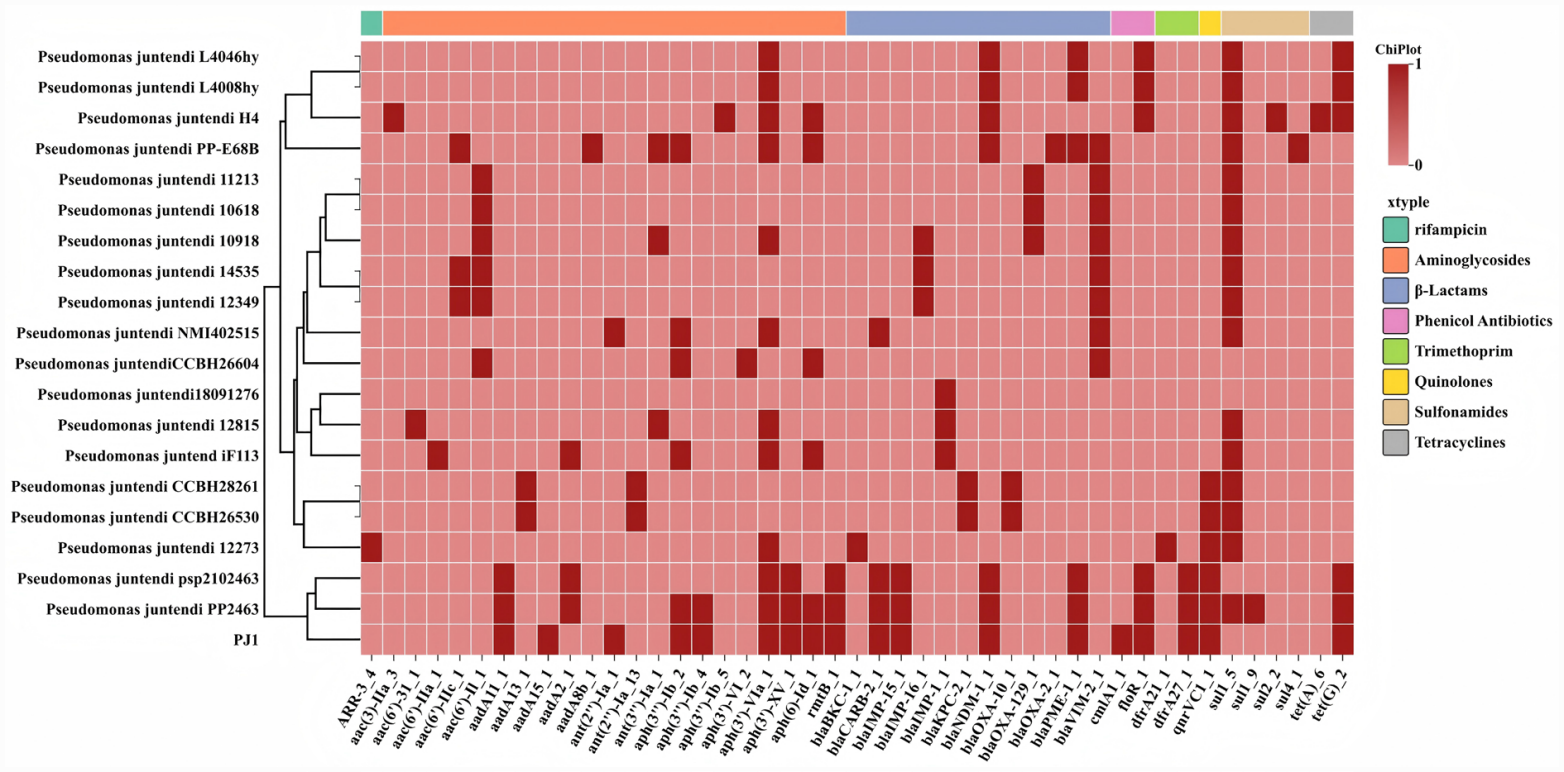
Heat map comparing the drug resistance genes carried by PJ1 and all clinical isolates of *P. juntendi* in NCBI.

Based on the antimicrobial susceptibility profiles shown in Table 1, we further compared the resistance gene repertoire of PJ1 with those of all previously reported clinical *P. juntendi* isolates available in the NCBI database (Figure 3). The heat map revealed that clinical isolates commonly harbor resistance genes from multiple antimicrobial classes, although the combinations of these genes varied markedly among different strains. PJ1 harbored multiple *β*-lactamase genes, including *bla*_NDM-1_, *bla*_IMP-15_, *bla*_CARB-2_, and *bla*_PME-1_. Among them, *bla*_NDM-1_ and *bla*_IMP-15_ jointly conferred high-level resistance to carbapenems and cephalosporins, while *bla*_CARB-2_ and *bla*_PME-1_ were associated with resistance to carbenicillin and monobactams. In addition, PJ1 carried several non–β-lactam resistance genes, including aminoglycoside-modifying enzyme genes (*aadA11*, *aph(3′)-Ib*, *aph(3′)-VI*, and *rmtB*), chloramphenicol resistance genes (*cmlA1* and *floR*), folate pathway inhibitors resistance genes (*dfrA27*), sulfonamide resistance genes (*sul1* and *sul2*) and the tetracycline efflux pump *tet(G)*.

### 3.3 Genetic localization and comparative analysis of key resistance genes

**Figure 4:**
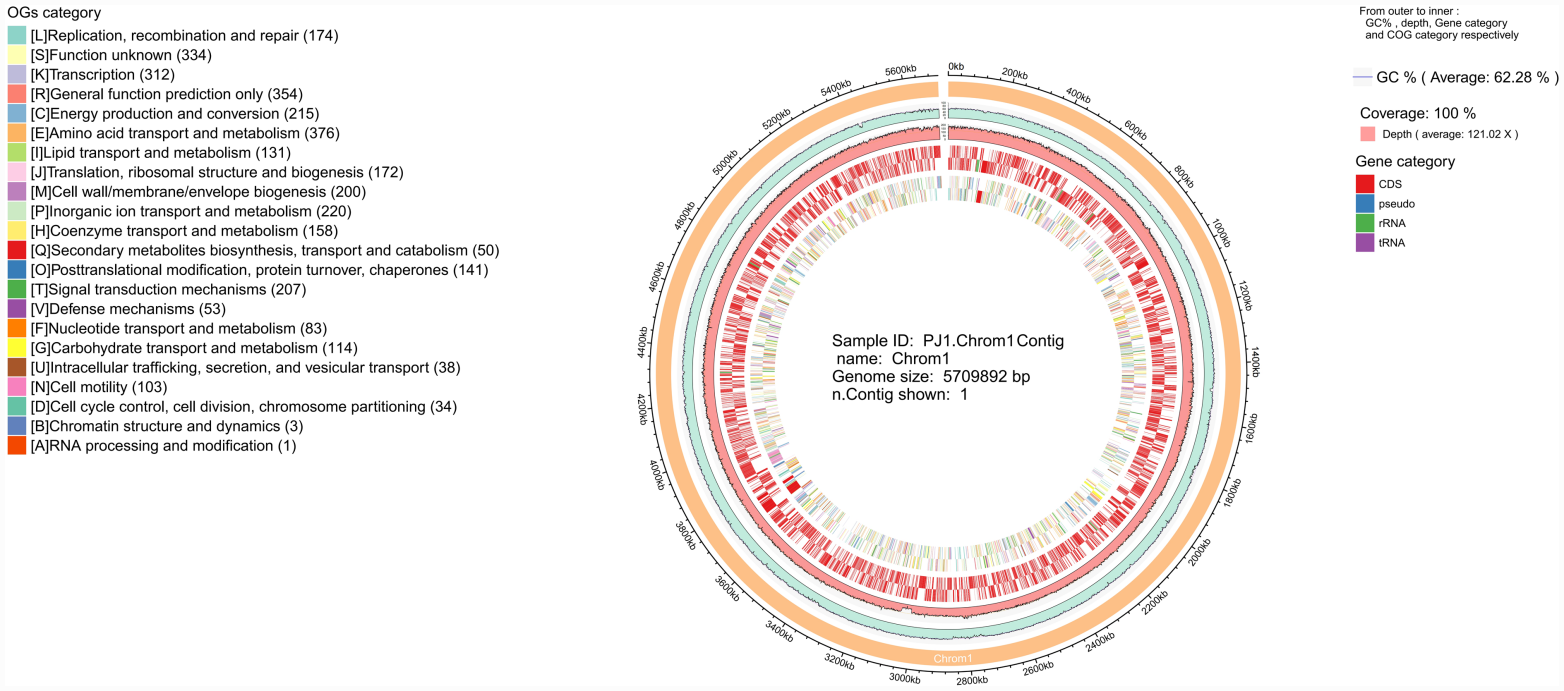
Circular representation of the PJ1 chromosome generated using Circos, showing GC content, sequencing depth, genomic features, and COG functional categories.

Following species identification of PJ1, we further analyzed the overall chromosomal genomic features of this isolate (Figure 4). The circular genome map shows that the complete chromosome of PJ1 comprises 5,709,892 bp with an overall GC content of 62.28%. The sequencing data achieved 100% coverage with an average depth of 121×, indicating a high-quality genome assembly.

**Figure 5:**
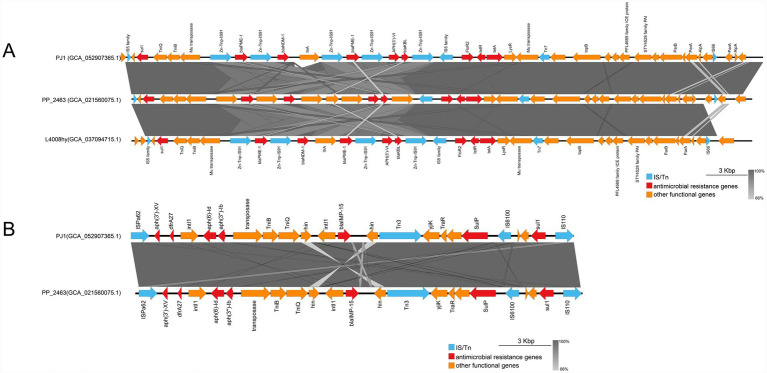
Linear comparison of resistance regions on the PJ1 chromosome: **(A)** Genetic context of *bla*_NDM-1_ and *bla*_PME-1_; **(B)** genetic context of *bla*_IMP-15_.

The *bla*_NDM-1_ gene is located on a ~ 45-kb composite multidrug-resistance (MDR) island on the chromosome (Figure 5A). Upstream of this region, multiple copies of *IS5* family insertion sequences are present, together with *sul1*, an AraC-family regulator, and Mu-type transposases, forming a structurally complex leading segment. In the core region, *bla*_PME-1_, *bla*_NDM-1_, and a second *bla*_PME-1_ appear in a “PME-NDM-PME” arrangement and are interspersed with several *IS91* domain proteins and a group II intron, constituting a highly repetitive and rearrangement-prone resistance module. Downstream, additional resistance determinants-including *aph(3′)-VIa*, *bla*_KBL_, *floR2*, *tetR(G)*, and *tet(G)-*coexist with multiple hypothetical proteins, transposition-related proteins, and island-maintenance elements such as *Tn7* transposase components, ParA/ParB partitioning systems, and the AlpA regulator. This configuration confers both composite resistance features and potential mobility. Overall, the NDM-associated region appears to be a chimeric MDR island formed through the accumulation of diverse transposable elements, intron components, and genomic-island modules, demonstrating marked genetic plasticity and a complex evolutionary trajectory.

Compared with the NDM module, the region harboring *bla*_IMP-15_ is situated within a ~ 19-kb integron-associated resistance island on the chromosome and exhibits features characteristic of a *Tn402-*like class 1 integron (Figure 5B). Upstream, the segment begins with an *IS110* family transposase, followed by *aph(3′)-XV, dfrA27*, and *intI1*, together with an *attI* site forming a complete integron platform. This is immediately succeeded by multiple aminoglycoside-modifying enzyme genes, recombinases, and components of the transposition complex such as TniB and TniQ, which flank the integron core. Within this structural context, *bla*_IMP-15_ is positioned between upstream and downstream recombinase-related elements. The downstream region connects to a *Tn3* family transposase, an *IS6100*-like transposase, and several proteins associated with stress response or genomic island maintenance, and terminates with *sul1* and another *IS110* family transposase, forming a characteristic “integron-*Tn3926-IS6100*” composite arrangement.

As shown in Figure 5, PJ1 and CP091088 share a highly conserved and collinear genetic backbone in the resistance regions carrying *bla*_NDM-1_, *bla*_PME-1_, and *bla*_IMP-15_, and they show almost identical arrangements of the core resistance genes and the surrounding IS and transposon elements. Only very small differences were found between the two isolates. PJ1 contains a CDS annotated as a group II intron reverse transcriptase and maturase in the *bla*_NDM-1_ and *bla*_PME-1_ cluster, while the same position in CP091088 is annotated as a hypothetical or shorter CDS. These minor differences do not change the overall structure of the resistance modules.

### 3.4 Biological phenotypes and virulence characteristics

**Figure 6:**
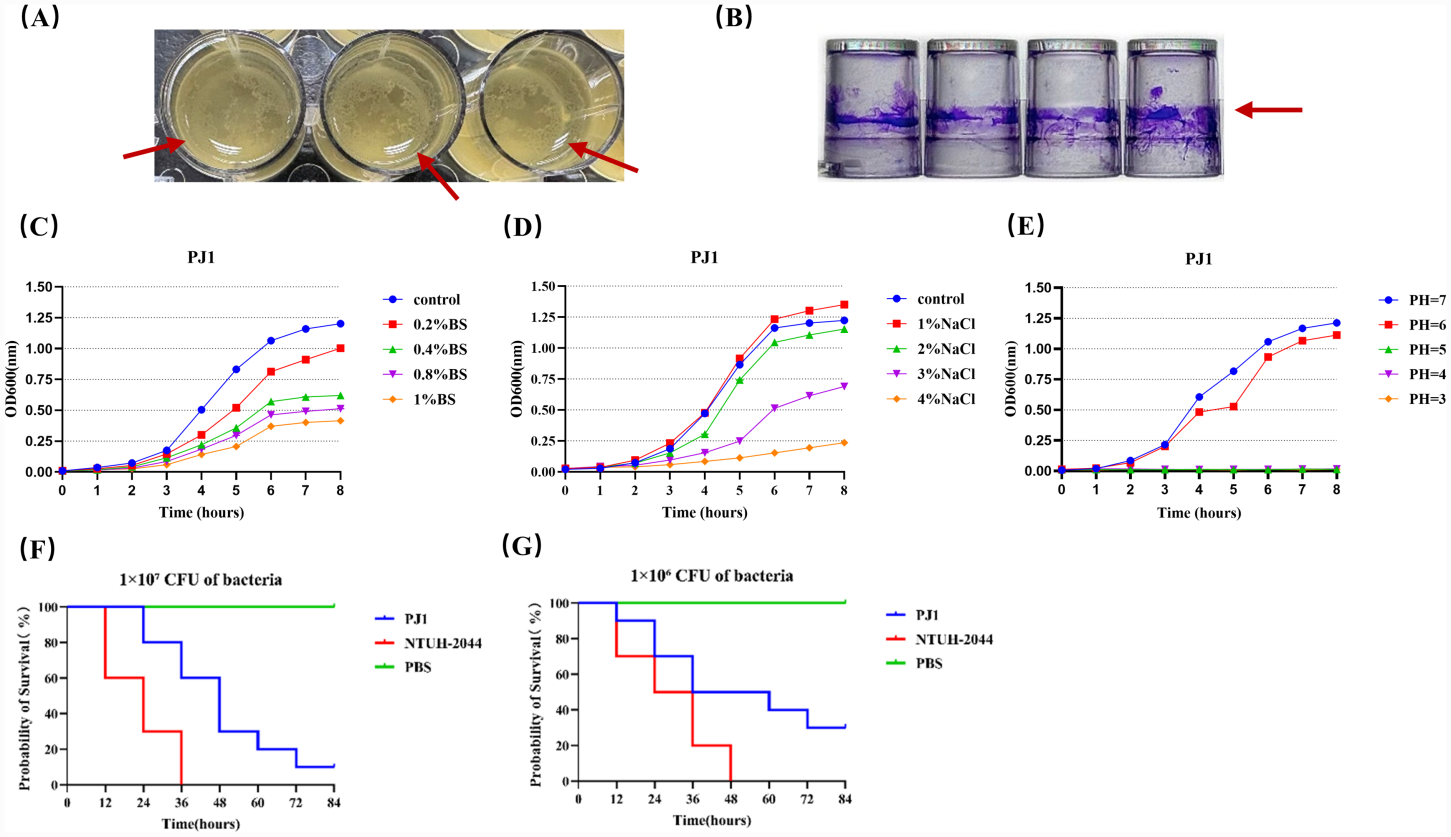
**(A)** Top view of the pellicle formed by *P. juntendi* strain PJ1 after 24 h of static incubation. **(B)** Biofilm formation of *P. juntendi* PJ1 after 24 h was observed by crystal violet assay. **(C–E)** Growth curves of *P. juntendi* PJ1 in different bile salt environments, different NaCl environments, and different acidic environments. **(F,G)** Survival curve of *G. mellonella* bacterial infection. Positive control group: hvKP NTUH-K2044; negative control group: PBS.

After 24 h of static incubation, PJ1 formed a visible pellicle structure on the liquid surface (Figure 6A), although the pellicle appeared relatively loose. Crystal violet staining confirmed a stable biofilm-forming ability of PJ1 (Figure 6B), with a mean OD₅₇₀ value of 2.89 ± 0.76, which was markedly higher than that of the blank control (OD₅₇₀ ≈ 0.038), indicating strong adhesive capacity and colonization potential. This phenotype was consistent with the genomic annotation of genes involved in flagellar assembly, type IV pili, and alginate biosynthesis, indicating that PJ1 possesses a well-developed adhesion and quorum-sensing regulatory system.

Growth curve analysis under different stress conditions (Figures 6C–E) showed that PJ1 maintained considerable growth activity in the presence of 1% bile salts and 3% NaCl, demonstrating high tolerance to bile and osmotic stress. Genomic analysis further revealed that PJ1 harbors complete RND, MFS, and ABC efflux systems, as well as numerous heavy metal resistance genes related to Zn, Cu, Fe, Ni, As, and Cd, together with tolC and acrAB systems associated with bile salt efflux. These molecular features collectively suggest that PJ1 possesses robust growth capacity and environmental adaptability under host-associated stress conditions.

Results from the *Galleria mellonella* infection model demonstrated that PJ1 caused larval death at both 1 × 10⁶ and 1 × 10^7^ CFU infection doses. The survival curves of infected larvae showed a significant decline compared with the PBS negative control, while remaining slightly less virulent than the hypervirulent *Klebsiella pneumoniae* NTUH-K2044 strain (Figures 6F,G). Genomic functional annotation revealed that PJ1 carries multiple virulence-associated determinants, including siderophore synthesis and uptake systems (pyoverdine, FbpABC, HitABC), type II and type VI secretion systems (T2SS, T6SS), lipopolysaccharide modification genes, and the two-component regulatory system PhoP/PhoQ. Together, these features constitute a potential virulence network contributing to its pathogenicity.

## 4 Discussion

This study reports a *Pseudomonas juntendi* strain (PJ1) isolated from a urinary tract infection case in Wuhu, Anhui Province, China. The genomic and phenotypic features of PJ1 highlight the potential clinical significance and adaptive capacity of this emerging species. Among the 67 publicly available *P. juntendi* genomes, 28 originate from human clinical samples, making humans the predominant host source identified so far. These isolates were recovered across multiple continents, primarily China, Japan, and Brazil, and span diverse temporal and ecological contexts. Urine remains the most common clinical source, whereas bloodstream infections appear particularly prevalent in Brazil. Additional isolates from sputum, stool, and wound samples suggest that *P. juntendi* may also colonize or infect the respiratory and gastrointestinal tracts.

Phylogenomic analysis further showed that human-derived isolates do not form a single host-associated lineage but instead cluster into multiple clades intermingled with animal- and environment-derived isolates, supporting the possibility of cross-host transmission among humans, animals, and environmental reservoirs. Notably, PJ1 clusters most closely with a urinary isolate from Sanmen County, China (GCA_024107335.1), forming a well supported lineage that also includes a porcine vaginal isolate from Hunan Province, China, and an animal intestinal isolate from Portugal. The presence of closely related strains across different hosts and geographic regions suggests a disseminating lineage with cross-host ecological flexibility. Recent genomic studies have similarly documented *P. juntendi* strains carrying carbapenemase genes within integrative conjugative elements or mosaic resistance islands, reinforcing the species’emerging epidemiological relevance (Zheng et al., 2022). Because PJ1 and related isolates can be misidentified as *P. putida* by routine MALDI-TOF MS workflows, their true prevalence and clinical impact may be underestimated, underscoring the need for genomic confirmation in surveillance programs (Liu et al., 2025).

The composite resistance islands carrying *bla*_NDM-1_ and *bla*_IMP-15_ on the PJ1 chromosome show evidence of multiple integration events and prolonged genomic rearrangements, features that are rare among nonclassical *Pseudomonas* species and indicate substantial genomic plasticity in this strain (Figure 5). The repeated combination of *IS5*, *IS91*, Mu-like transposases, and a group II intron within the NDM region indicates that this segment likely experienced multiple rounds of rolling-circle transposition, intron-mediated amplification, and transposon insertion during its formation (Janvier et al., 2013; Qamar et al., 2025). Notably, the PME–NDM–PME tripartite arrangement is absent from the typical *Tn125* platform, and its presence likely reflects the stepwise assembly, expansion, and functional integration of resistance modules originating from different sources into the chromosome (Benigno et al., 2023).

The IMP region presents a distinct and highly modular resistance island architecture. Centered on a class 1 integron, it incorporates the *attI* site, recombinases, the TniQ and TniB complex, *Tn3* elements, and *IS6100*, together forming an integrated integron–*Tn3–IS6100* framework. Such configurations are generally considered to enhance both the mobility and the stability of associated resistance genes (Wang et al., 2019). The integron plays a central role in clustering IMP and various non beta lactam resistance genes, enabling this region to rapidly acquire, replace, or amplify functional gene cassettes under selective pressure, thereby providing the genetic basis for swift adaptation of resistance (Bhat et al., 2025).

The coexistence of these two resistance modules indicates that PJ1 has acquired *bla*_NDM-1_ and *bla*_IMP-15_ through different chromosomal integration processes, with the *bla*_NDM-1_ module being located in an *ICE*-associated composite resistance island and the *bla*_IMP-15_ module being embedded in an integron-associated *Tn402*-like region. As a result, the PJ1 chromosome contains a mosaic multidrug resistance island composed of multiple mobile genetic elements. This type of chromosomal organization is still rare among Pseudomonas species but agrees with recent reports of *P. juntendi* strains carrying more than one carbapenemase gene (Zheng et al., 2022; Jiang et al., 2024; Alberto-Lei et al., 2022).

At the phenotypic level, PJ1 demonstrated strong environmental adaptability and colonization capacity, including the ability to form stable biofilms under static conditions, sustain vigorous growth under multiple stresses, and exhibit measurable virulence in the *Galleria mellonella* infection model (Figures 6A–G). To better interpret these traits from a genomic perspective, we examined the functional composition of the PJ1 genome through GO, KEGG, and COG analyses. GO terms were enriched for genes involved in metabolism, transport, cellular processes, and stress responses, with substantial representation of membrane-associated components and protein complexes, suggesting enhanced capacities for nutrient acquisition, transmembrane transport, and stress sensing (Supplementary Figure S3). KEGG pathway annotations further revealed complete metabolic networks, including carbon, amino acid, and energy pathways, along with an overrepresentation of genes mediating membrane transport and signal transduction, consistent with the strain’s ability to withstand osmotic, nutrient, and environmental fluctuations (Supplementary Figure S4). COG categories linked to replication, recombination, repair, transcriptional regulation, and cell envelope biogenesis were also prominently represented, reflecting a high degree of genomic plasticity and regulatory versatility (Supplementary Figure S2). Together, these functional signatures provide a genomic basis for PJ1’s biofilm formation, stress tolerance, and ecological persistence.

From a public health standpoint, the genomic features of PJ1 raise important concerns regarding the persistence and dissemination of carbapenemase determinants in clinical and environmental settings. The strain carries two independently assembled chromosomal MBL platforms NDM-1 and IMP-15, each stabilized by integron-, *IS91*-, or *ICE*-associated modules, providing multiple genetic pathways for long-term maintenance and potential horizontal exchange of resistance genes (Zhao et al., 2025). Combined with PJ1s demonstrated biofilm formation, stress tolerance, and heavy-metal resistance, these features suggest that the species is well equipped to survive in hospital wastewater systems and other selective microenvironments where MBL-producing *Pseudomonas* are known to persist (Tada et al., 2016). Misidentification of *P. juntendi* as *P. putida* by MALDI-TOF MS further increases the risk of unrecognized circulation. Incorporating genomic ANI-based identification and targeted screening of *ICE/IS91*-associated mobile elements into routine surveillance will be essential to delineate the ecological routes through which *P. juntendi* contributes to the regional and potentially broader dissemination of carbapenemase genes.

## 5 Conclusion

This study identifies PJ1 as a multidrug-resistant *Pseudomonas juntendi* strain carrying two chromosomally integrated MBL determinants, *bla*_NDM-1_ and *bla*_IMP-15_. Both resistance islands exhibit highly mosaic architectures shaped by integron-, *IS91*-, and *ICE*-associated elements, reflecting substantial genomic plasticity. The strains’ cross-host lineage background and strong adaptive traits suggest a potential role for *P. juntendi* as a reservoir for carbapenemase genes in clinical and environmental settings. Enhanced genomic identification and targeted surveillance will be essential to monitor its dissemination and public health impact in One Health settings.

## Data Availability

The datasets presented in this study can be found in online repositories. The names of the repository/repositories and accession number(s) can be found at: https://www.ncbi.nlm.nih.gov/, GCA_052907365.1.
