# Supplementary figures and images for: First report of a urinary Pseudomonas juntendi carrying blaNDM-1 and blaIMP-15 co-integrated into the chromosome via ICE-IS91 and integron-Tn402-like transposition modules
Source: Front Microbiol. 2026 Jan 29;17:1724958. doi: 10.3389/fmicb.2026.1724958 (PMC12894362; doi:10.3389/fmicb.2026.1724958)

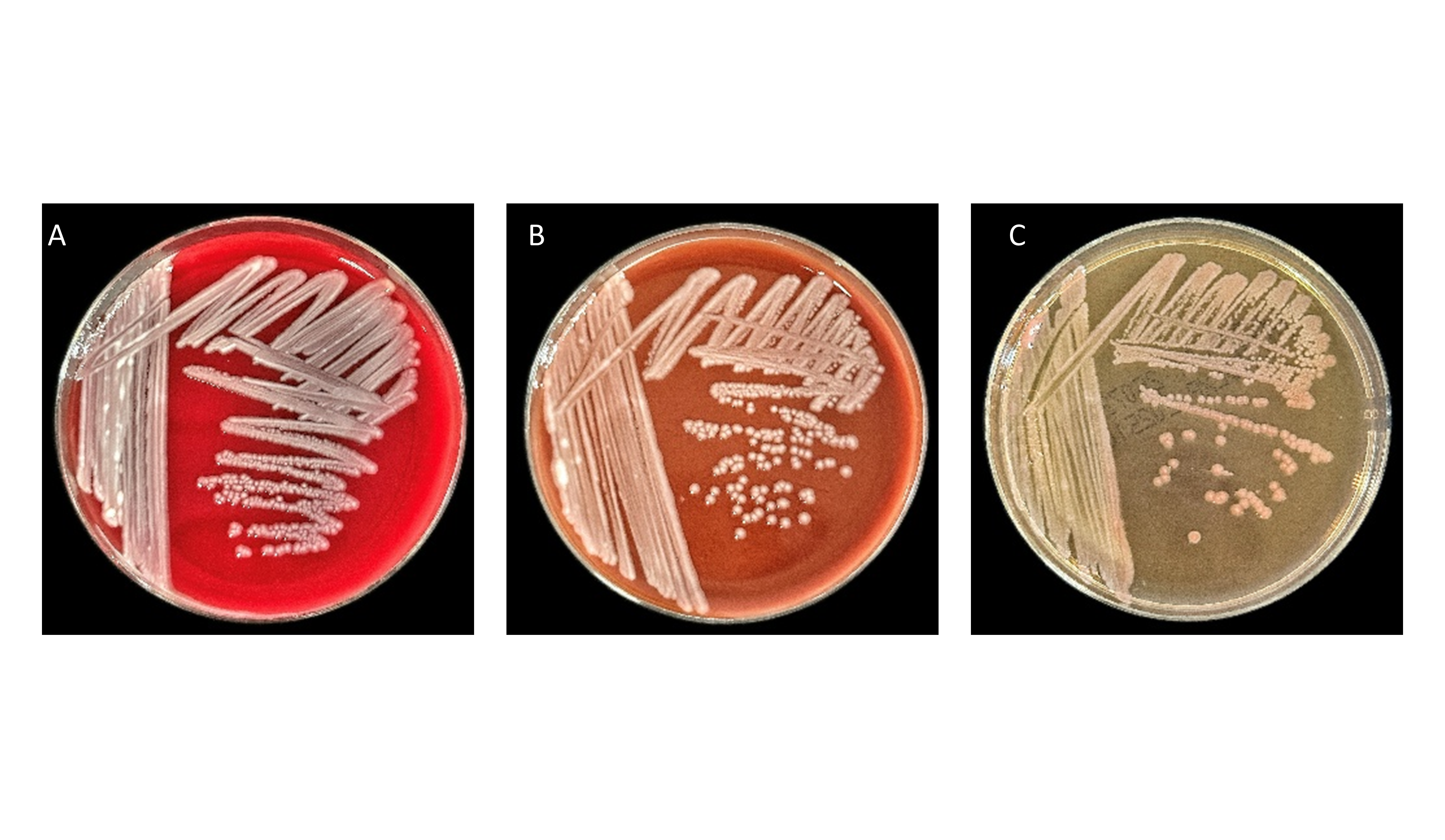

Supplement: Supplementary file 1 [file Image_1.TIF]

Gene Function Classification (GO)

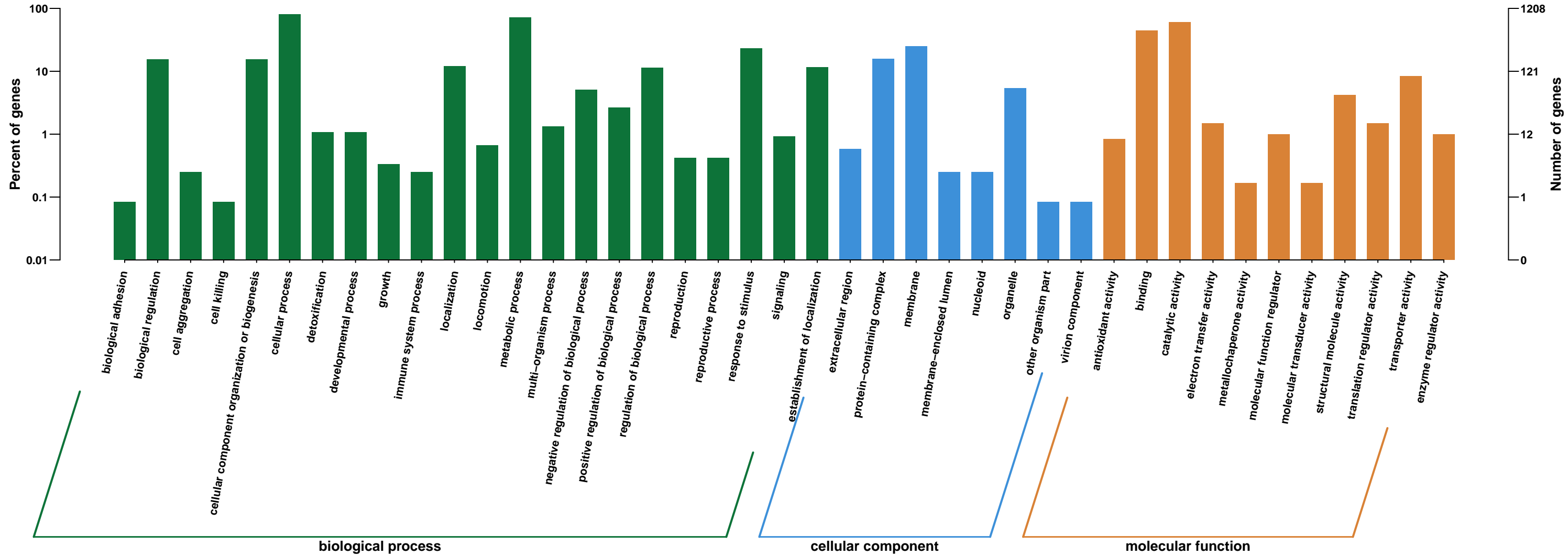

Supplement: Supplementary file 3 [file Image_3.PDF]

# KEGG Pathway Classification

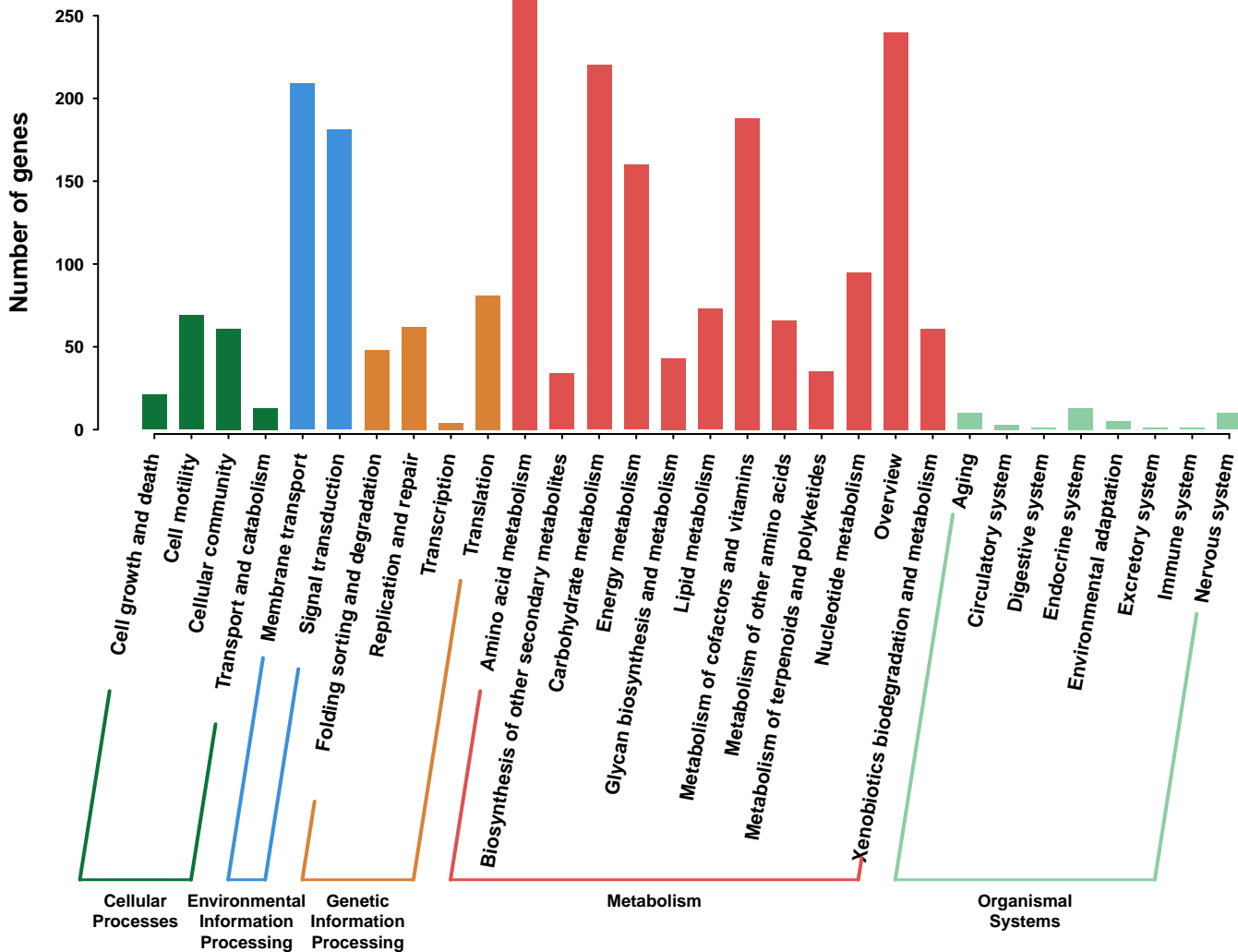

Supplement: Supplementary file 4 [file Image_4.PDF]

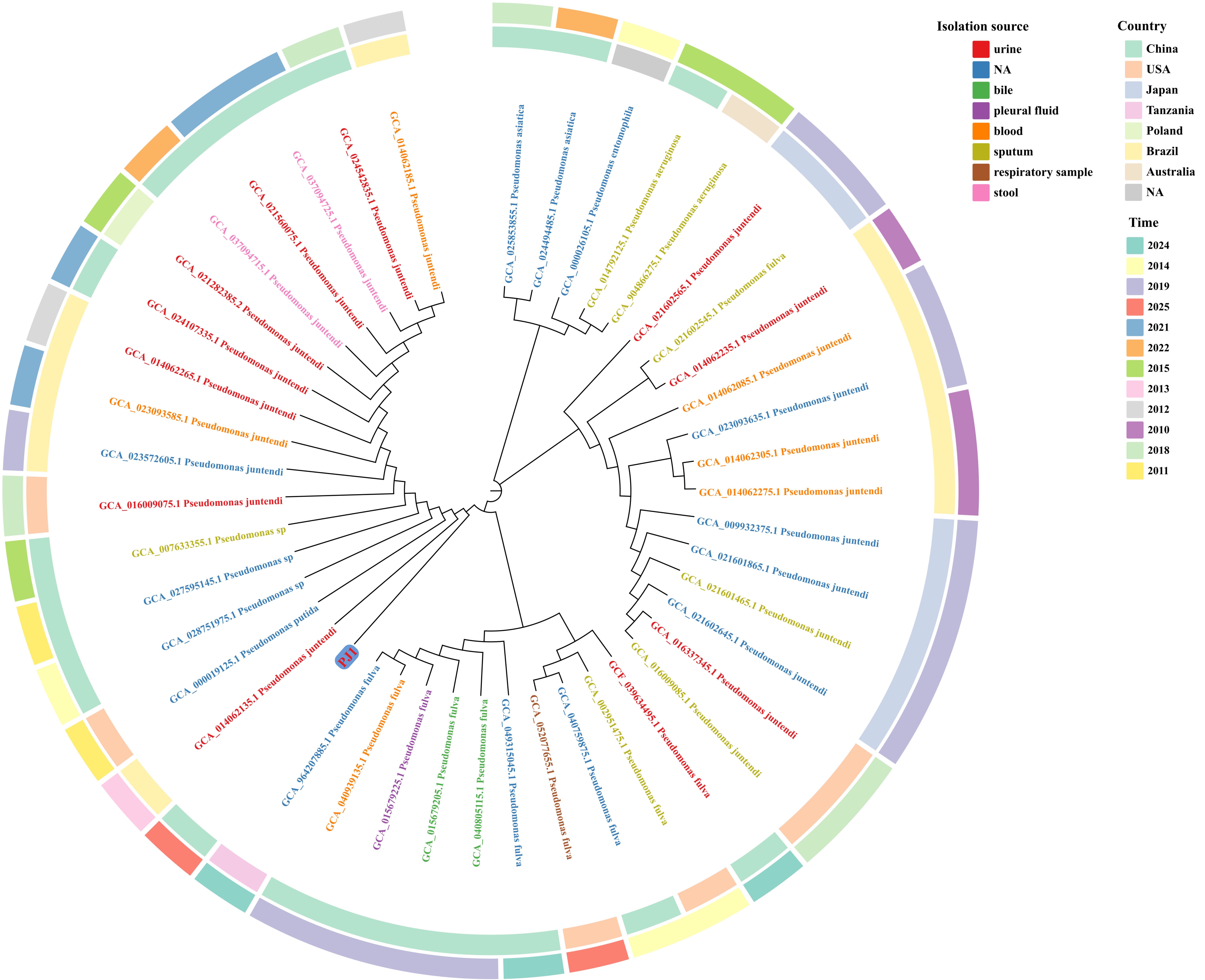

Supplement: Supplementary file 5 [file Image_5.PDF]
