# Supplementary material for: First report of a urinary Pseudomonas juntendi carrying blaNDM-1 and blaIMP-15 co-integrated into the chromosome via ICE-IS91 and integron-Tn402-like transposition modules
Source: Front Microbiol. 2026 Jan 29;17:1724958. doi: 10.3389/fmicb.2026.1724958 (PMC12894362; doi:10.3389/fmicb.2026.1724958)
Supplement: Supplementary file 2 [file Image_2.PDF]

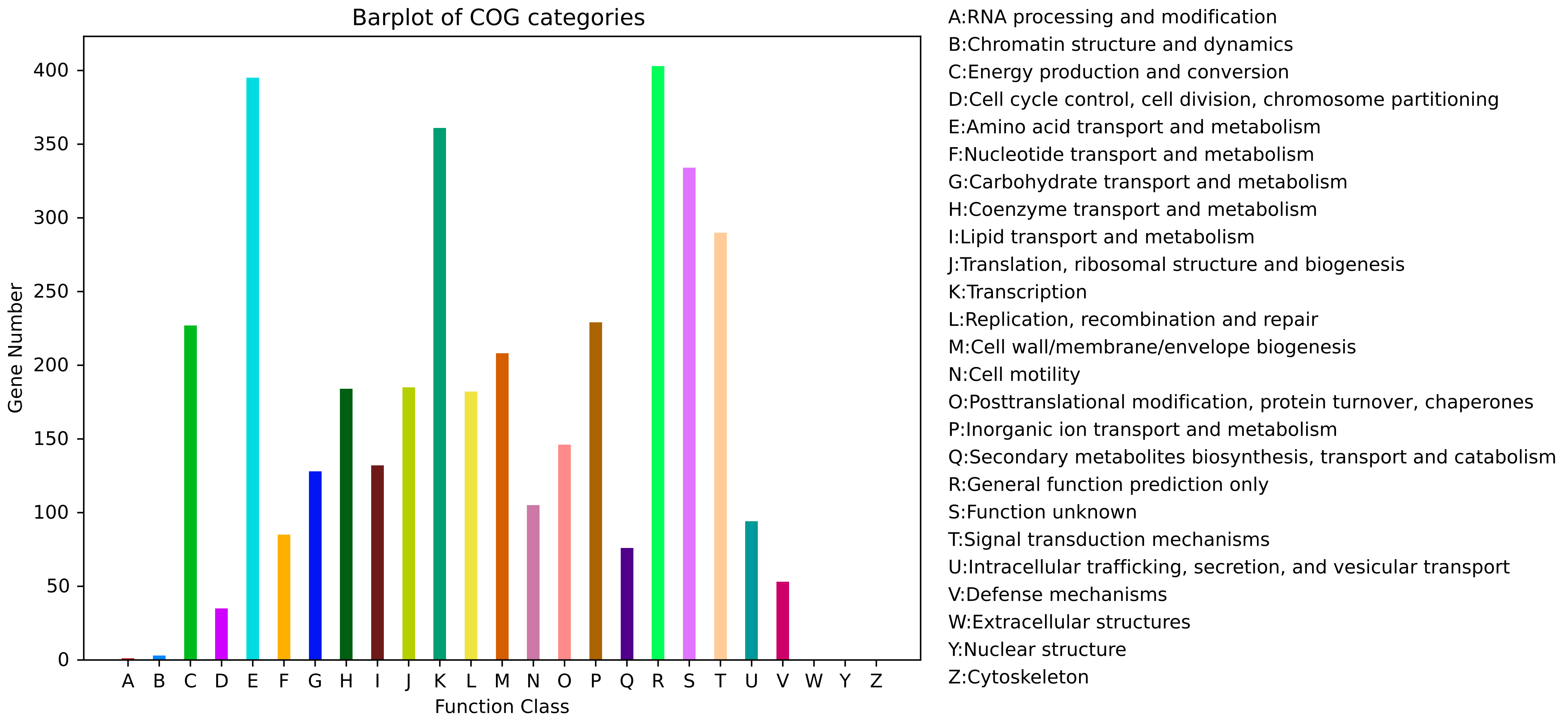
Barplot of COG categories
Gene Number
0
50
100
150
200
250
300
350
400
A B C D E F G H I J K L M N O P Q R S T U V W Y Z
Function Class
A:RNA processing and modification
B:Chromatin structure and dynamics
C:Energy production and conversion
D:Cell cycle control, cell division, chromosome partitioning
E:Amino acid transport and metabolism
F:Nucleotide transport and metabolism
G:Carbohydrate transport and metabolism
H:Coenzyme transport and metabolism
I:Lipid transport and metabolism
J:Translation, ribosomal structure and biogenesis
K:Transcription
L:Replication, recombination and repair
M:Cell wall/membrane/envelope biogenesis
N:Cell motility
O:Posttranslational modification, protein turnover, chaperones
P:Inorganic ion transport and metabolism
Q:Secondary metabolites biosynthesis, transport and catabolism
R:General function prediction only
S:Function unknown
T:Signal transduction mechanisms
U:Intracellular trafficking, secretion, and vesicular transport
V:Defense mechanisms
W:Extracellular structures
Y:Nuclear structure
Z:Cytoskeleton
